# Population-Level Trends in Asthma Exacerbations After Introduction of Respiratory Biologics

**DOI:** 10.1001/jamanetworkopen.2026.20272

**Published:** 2026-06-24

**Authors:** Yung-Fang Tu, David W. Stein, Ayobami Akenroye

**Affiliations:** 1 Department of Biostatistics, Harvard T.H. Chan School of Public Health, Boston, Massachusetts; 2 Center for Clinical Investigation, Department of Medicine, Brigham and Women’s Hospital, Boston, Massachusetts; 3 Harvard Medical School, Boston, Massachusetts; 4 Division of Allergy and Clinical Immunology, Department of Medicine, Brigham and Women’s Hospital, Boston, Massachusetts; 5 Channing Division of Network Medicine, Department of Medicine, Brigham and Women’s Hospital, Boston, Massachusetts

## Abstract

**Importance:**

Respiratory biologics have transformed the treatment for asthma, a disease affecting approximately 300 million individuals globally.

**Objective:**

To evaluate population-level trends in asthma exacerbations, including during a period encompassing approval of multiple biologics.

**Design, Setting, and Participants:**

This cohort study used electronic health records from a large health care system in Boston, Massachusetts, including specialty allergy and pulmonology clinics, from January 2006 to May 2025. Adults aged 18 years or older with physician-diagnosed asthma seen in specialty clinics were included. Patients with other chronic lung diseases, except chronic obstructive pulmonary disease, were excluded.

**Exposure:**

The year 2015 was designated a priori as the exposure change point to assess changes in asthma exacerbations, given that it marked the beginning of a period of rapid approval of respiratory biologics with 4 of the 6 respiratory biologics approved between 2015 and 2018.

**Main Outcomes and Measures:**

The primary outcomes were the changes in the intercept and slope of annual exacerbations per 1000 asthma patients per year. Immediate and sustained reductions in exacerbations were evaluated using the interrupted time series analytic approach. Subgroup analyses evaluated changes by severity, baseline eosinophil count, and demographic or clinical characteristics.

**Results:**

Among 5269 adults with asthma (mean [SD] age, 51.9 [18.3] years; 3761 female [71.4%]), exacerbations increased from 2006 to 2015 (155.4 [95% CI, 117.7 to 193. 2] events per 1000 patients per year). After 2015, an immediate reduction occurred (–474.1 [95% CI, −783.2 to −165.0] events per 1000 patients per year), followed by a sustained yearly decline (–206.5 [95% CI, −259.9 to −153.0] events per 1000 patients per year). Patients with 2 or more annual exacerbations showed greater improvements (immediate: –808.2 [95% CI, −1646.5 to 30.2] events per 1000 patients per year; slope: –374.5 [95% CI, −519.4 to −229.7] events per 1000 patients per year) than patients with fewer than 2 exacerbations. Those with baseline eosinophil levels of 450 cells/μL or greater exhibited significant declines (immediate: –1799.8 [95% CI, −3555.3 to −44.3] events per 1000 patients per year; slope: –443.4 [95% CI, −746.7 to −140.0] events per 1000 patients per year) compared with patients with eosinophil levels less than150 cells/μL. Reductions occurred across age, sex, body mass index, smoking, and insurance subgroups, although smokers and inner-city residents retained higher absolute rates.

**Conclusions and Relevance:**

In this cohort study of 5269 adults with asthma, there were significant reductions in asthma exacerbations, particularly among patients with severe or eosinophilic disease. Importantly, these benefits closed the outcome gap between patients with moderate to severe asthma and those with mild asthma, and support the effectiveness of biologics, highlighting their potential to improve outcomes among high-risk populations.

## Key Points

**Question:**

Among adults with physician-diagnosed asthma in an integrated health system, what is the association of the introduction of biologics with population-level asthma exacerbation events?

**Findings:**

This cohort study of 5269 adults found that asthma exacerbation events declined significantly immediately after 2015, with sustained yearly decreases. Patients with more severe disease and elevated eosinophil counts experienced the largest reductions.

**Meaning:**

These findings suggest that introduction of respiratory biologics for the treatment of moderate to severe asthma led to significant improvements in asthma outcomes, with reduction of the outcome gap between these patients and patients with mild asthma, who would usually not be eligible for these therapies.

## Teaser

This cohort study analyzes population-level changes in asthma exacerbations after the rapid approval of respiratory biologics in 2015.

## Introduction

Asthma is a global disease with substantial morbidity and mortality.^1^ Patients with severe asthma bear a disproportionate share of its morbidity and mortality.^2,3^ For these patients, the approval of monoclonal antibodies (biologics) over the past 1 to 2 decades has been a great alternative to systemic corticosteroids given the significant risks associated with long-term use of systemic corticosteroids, including adrenal suppression, neuropsychiatric adverse events, and osteoporosis.^4,5,6,7^

The introduction of these biologic therapies as adjunctive therapy in patients with severe asthma has transformed the treatment landscape. The first respiratory biologic, an anti-immunoglobulin E therapy, omalizumab, was approved in 2003;^8^ however, the next available asthma biologic, mepolizumab, was not approved until 2015. Since 2015, 5 more biologics have been approved, including 4 that were approved between 2015 and 2018, a time period we termed the respiratory biologics boom.^9,10^ In randomized trials, biologics were associated with patient-level reductions in exacerbations and reduction or elimination of the need for systemic corticosteroids.^11,12,13,14^ However, reported impact at the population level in clinical cohorts and disproportionately affected subgroups, such as by baseline exacerbation burden, is limited. In this study, we investigated the population-level changes in exacerbation trends before and after the onset of the respiratory biologics boom in 2015.

## Methods

This cohort study was approved by the Mass General Brigham institutional review board with a waiver of informed consent due to the use of preexisting retrospective data with minimal risk in accordance with the Common Rule. Reporting follows the Strengthening the Reporting of Observational Studies in Epidemiology (STROBE) reporting guideline for cohort studies.

### Data Source and Population

We included patients aged 18 years and older with physician-diagnosed asthma seen between January 1, 2006, and May 31, 2025, in the allergy and/or pulmonology specialty clinics of a large integrated health care system in Boston, Massachusetts. We included all patients with the *International Classification of Diseases, Ninth Revision (ICD-9) *or *International Statistical Classification of Diseases and Related Health Problems, Tenth Revision (ICD-10)* codes for asthma (493.x and J45.x respectively) regardless of whether they were biologic-naive or receiving a respiratory biologic. We excluded individuals with other chronic lung diseases including interstitial lung disease, cystic fibrosis, and bronchiectasis. We did not exclude patients with concomitant chronic obstructive pulmonary disease (COPD), a relatively common comorbidity with asthma. Baseline covariates potentially associated with biologic utilization and/or effectiveness were extracted, including demographic characteristics, laboratory variables such as the maximum blood eosinophil count (BEC) within a 3-year window, and baseline asthma severity. Race and ethnicity were included as a self-reported demographic variable. Race categories included Asian, Black, White and other (American Indian or Alaska Native, Native Hawaiian or Other Pacific Islander, 2 or more races, or any race not otherwise specified, as well as those who declined to report their race or had missing or unknown race information), and ethnicity categories included Hispanic, non-Hispanic, and other (participants who declined to report their ethnicity or had missing or unknown ethnicity information). Race and ethnicity were included to account for a known disparity in the prescription and use of biologic therapies by race and ethnicity.

### Main Outcomes and Measures

The primary outcomes were the changes in the intercept and slope of annual exacerbations per 1000 patients for each calendar year. We defined exacerbations as an acute worsening of asthma that required an emergency department visit, hospital admission, or a prescription of 3 to 28 days of oral corticosteroid therapy. Events that occurred within 7 days of each other were considered the same event.^15^

### Construction of Annual Outcome Measures

For each patient, cohort entry (index date) was defined as the earliest date of a recorded *ICD-9* or *ICD-10* code for moderate to severe asthma. To construct population-level annual measures, exacerbation events were assigned to calendar years based on the index date. For index dates between January 1 and June 30 of a given calendar year, preindex exacerbations were attributed to the preceding calendar year and postindex exacerbations were attributed to the index year. For index dates between July 1 and December 31, preindex exacerbations were assigned to the index year and postindex exacerbations were assigned to the subsequent year. These preindex and postindex exacerbation counts were aggregated to estimate annual exacerbation rates at the population level for each calendar year.

### Statistical Analysis

Interrupted time series models were employed to estimate preexposure and postexposure trends.^16,17^ We employed a segmented multivariable linear regression model using the following covariates: (1) *t*, a continuous variable for time in years since the start of observation (*t* = 0 in 2006, *t* = 1 in 2007, and increasing annually thereafter); (2) *D*, a binary indicator for postexposure period (D = 0 for 2006-2014 and *D* = 1 for 2015-2025); and (3) timepost, a continuous variable for time since exposure (timepost = 0 before 2015, timepost = 1 in 2015, and increasing annually thereafter). This model enabled estimation of baseline trends, immediate changes after the exposure, which represents a level change at the first post-2015 time point and reflects differences in annual exacerbation rates per 1000 patients during the first year following the intervention period, and postexposure trend changes (change in slope).^16,17^

We conducted subgroup analysis by demographic and clinical characteristics to assess potential heterogeneity in treatment effects. This included by age (18-39, 40-64, and ≥65 years), sex, body mass index (BMI; calculated as weight in kilograms divided by height in meters squared), smoking status, residence in the inner city (a proxy for socioeconomic status based on the Metropolitan Area Planning Council designation of inner core), and insurance type. Given that BEC is a key biomarker for eligibility and response to respiratory biologics and because corticosteroid use could suppress the baseline BEC, we conducted a stratified analysis based on maximum BEC measured within 3 years prior to cohort entry. Patients were categorized into 4 groups (<150 cells/μL, 150-299 cells/μL, 300-449 cells/μL, and ≥450 cells/μL). To further support attribution of observed population-level changes to biologic therapies and because biologic eligibility is typically limited to patients with severe, uncontrolled asthma, we conducted subgroup analysis based on disease severity: 2 or more exacerbations at baseline compared with patients with fewer than 2 exacerbations. To address potential misclassification in *ICD-9* or *ICD-10* coding, we performed a similar analysis stratified by use of maintenance therapies in addition to inhaled corticosteroids (ICS). We hypothesized that the group with worse disease severity would show significant immediate and sustained improvements in asthma exacerbations post-2015 given that this is the group that would typically be eligible for biologic therapy. Those with fewer than 2 exacerbations at baseline and those who did not require additional maintenance therapies beyond ICS would show no significant reductions in exacerbations post-2015.

In sensitivity analyses, we used inverse probability treatment weighting to address potential confounding from covariate imbalance between the pre-2015 and post-2015 periods. Covariates were selected via penalized (least absolute shrinkage and selection operator) logistic regression with 10-fold cross-validation. Variables with nonzero coefficients at the 1-SE penalty were included in the inverse probability treatment weighting model. Inverse probability weights were truncated at the first and 99th percentile to limit the influence of extreme values.^18,19^ Second, we adjusted for the potential reductions in exacerbation rates due to the COVID-19 pandemic and the uptake of synchronized maintenance and reliever therapy (SMART) with ICS-formoterol, recommended in 2020 by the National Asthma Education and Prevention Program and aligned with the Global Initiative for Asthma.^20,21^ Postexposure outcomes were modeled in 2 segments: the pre–COVID-19 and pre-SMART period (2015-2019) and the post–COVID-19 and post-SMART period (2020-2025). Based on prior estimates, we assumed a 30% to 50% reduction in exacerbations. Thus, we accordingly inflated observed exacerbations by 30% and 50% in the 2020 to 2025 period and compared this with the original observed outcome.^22^ Lastly, to account for autocorrelation and potential heteroskedasticity in the residuals in our original model, we fitted a generalized least-squares regression model with an autoregressive correlation structure of order 3, allowing residuals to be correlated across a 3-year span. The 3-year window was selected based on the results of Breusch-Godfrey test and Akaike information criterion.^23^ A 2-sided *P* < .05 was considered statistically significant for all analyses. All analyses were conducted in R.4.4.1 (R Project for Statistical Computing).

## Results

### Characteristics of Study Population

A total of 5269 patients were included in this study. Among the cohort, the mean (SD) age was 51.9 (18.3) years, 3761 (71.4%) were female, 14 (2.7%) identified as Asian, 484 (9.2%) identified as Black, 152 (2.9%) identified as Hispanic, and 3920 (74.4%) identified as White. Among all participants, 3289 (62.4%) were covered by private insurance (Table 1).

**Table 1.** Baseline Characteristics of Study Participants

Characteristics	Participants, No. (%)^a^			SMD
	All (N = 5269)	Pre-2015	Post-2015	
Age, mean (SD), y	51.9 (18.3)	46.1 (17.4)	54.5 (18.2)	0.47
Sex				
Female	3761 (71.4)	1156 (75.9)	2414 (69.6)	0.14
Male	1508 (28.6)	368 (24.1)	1056 (30.4)	
Ethnicity				
Hispanic	152 (2.9)	18 (1.2)	127 (3.7)	0.98
Non-Hispanic	2707 (51.4)	315 (20.7)	2151 (62.0)	
Other^b^	2410 (45.7)	1191 (78.1)	1192 (34.4)	
Race				
Asian	142 (2.7)	36 (2.4)	93 (2.7)	0.09
Black	484 (9.2)	125 (8.2)	335 (9.7)	
White	3920 (74.4)	1176 (77.2)	2549 (73.5)	
Other^c^	723 (13.7)	187 (12.3)	493 (14.2)	
Body mass index, mean (SD)^d^	29.2 (7.7)	28.7 (8.1)	29.4 (7.5)	0.03
Smoking status				
Current or former	1117 (21.2)	379 (24.9)	738 (21.3)	0.09
Never or unknown	4152 (78.8)	1145 (75.1)	2732 (78.7)	
Insurance				
Public	1980 (37.6)	620 (40.7)	1279 (36.9)	0.08
Private	3289 (62.4)	904 (59.3)	2191 (63.1)	
Residence				
Inner city	724 (13.7)	149 (9.8)	540 (15.6)	0.18
Other	4545 (86.3)	1375 (90.2)	2930 (84.4)	
Comorbidities				
Chronic obstructive lung diseases	611 (11.6)	53 (3.5)	558 (16.1)	0.43
Chronic rhinosinusitis with or without nasal polyposis	414 (7.9)	87 (5.7)	327 (9.4)	0.14
Allergic rhinitis	3807 (72.3)	1171 (76.8)	2636 (76.0)	0.02
Atopic dermatitis	160 (3.0)	63 (4.1)	97 (2.8)	0.07
Charlson Comorbidity Index, mean (SD)	0.8 (1.4)	0.3 (0.7)	1.0 (1.6)	0.62
Annual exacerbation counts, mean (SD)	1.2 (2.0)	1.0 (2.1)	1.2 (2.0)	0.12
Medication				
ICS, ever	1488 (28.2)	396 (26.0)	1092 (31.5)	0.12
ICS or long-acting β-agonists, ever	3432 (65.1)	798 (52.4)	2634 (75.9)	0.49
Long-acting muscarinic agonists, in the past y	716 (13.6)	130 (8.5)	586 (16.9)	0.25
Leukotriene receptor agonists, in the past y	1458 (27.7)	353 (23.2)	1105 (31.8)	0.20

Abbreviations: ICS, inhaled corticosteroids; SMD, standardized mean difference.

^a^ There were 887 participants (16.8%) lost to follow-up.

^b^ Included participants who declined to report their ethnicity or had missing or unknown ethnicity information. This included individuals with missing ethnicity (34.6%).

^c^ Included participants who self-identified as American Indian or Alaska Native, Native Hawaiian or Other Pacific Islander, 2 or more races, or any race not otherwise specified, as well as those who declined to report their race or had missing or unknown race information.

^d^ Calculated as weight in kilograms divided by height in meters squared. There were 373 participants (7.1%) with missing values.

### Changes Immediately Following and After Exposure (2015)

**Figure 1.**
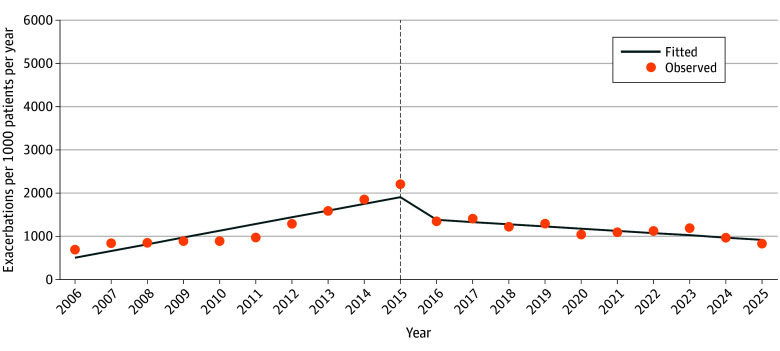
Line Graph of Interrupted Time Series Analysis of Exacerbation Events The dashed line at 2015 indicate the exposure change point to assess changes in asthma exacerbations.

Prior to 2015, there was an upward trend in annual exacerbation events (155.4 [95% CI, 117.7 to 193.2] exacerbation events per 1000 patients per year) (Table 2). Following 2015, we observed a significant immediate reduction in the annual exacerbation events (–474.1 [95% CI, −783.2 to −165.0] exacerbation events per 1000 patients per year) and a sustained improvement over time (–206.5 [95% CI, −259.9 to −153.0] exacerbation events per 1000 patients per year) in the postexposure period (Figure 1).

**Table 2.** Interrupted Time Series Model for Asthma Exacerbation Events Per 1000 Patients

Covariate	Events per 1000 patients per year (95% CI)	*P* value
Before 2015	155.4 (117.7 to 193.2)	<.001
Immediately following 2015	−474.1 (−783.2 to −165.0)	.005
After 2015	−206.5 (−259.9 to −153.0)	<.001

### Subgroup Analyses

#### By Age and Sex

**Figure 2.**
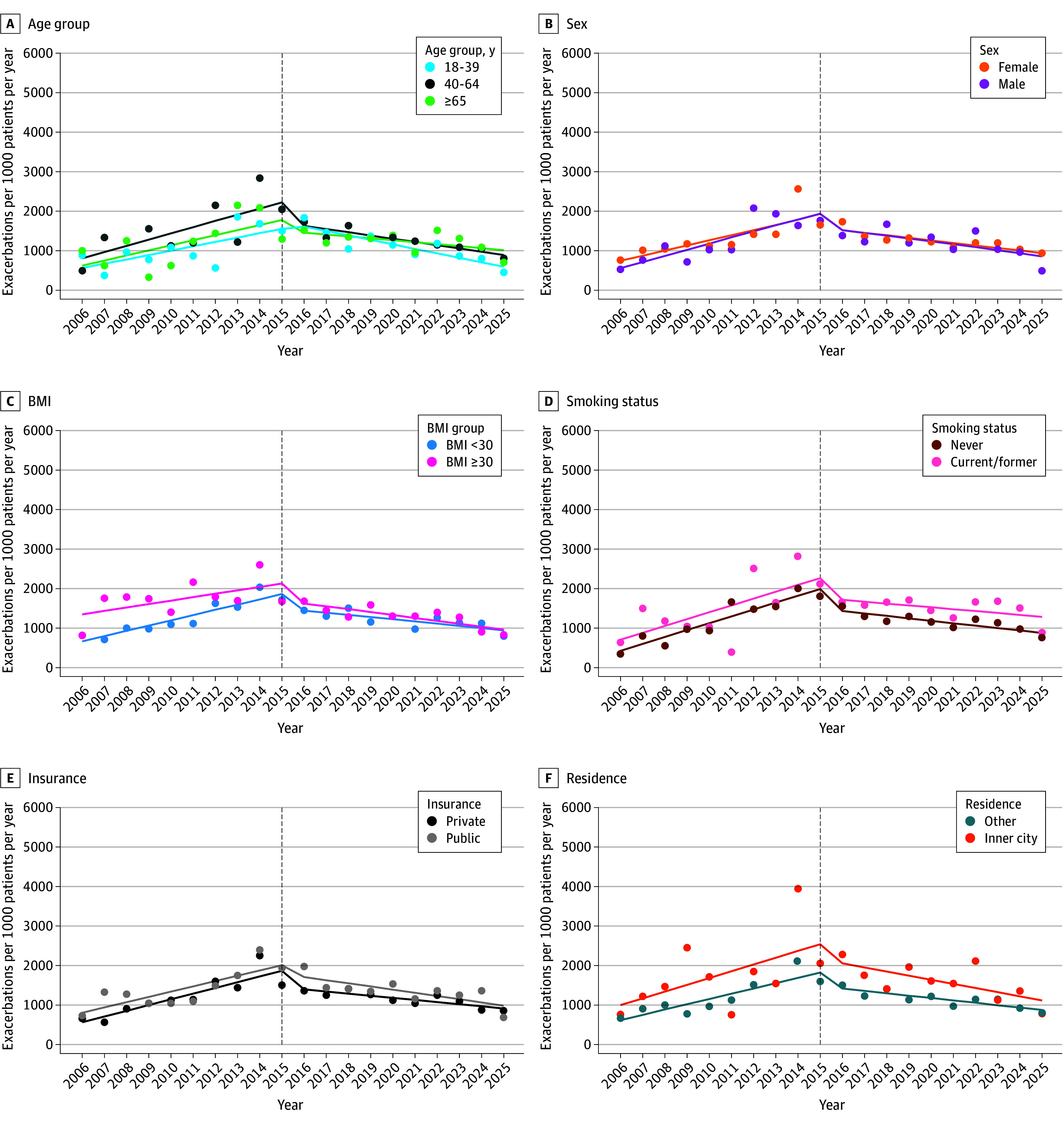
Line Graphs of Exacerbation Rates by Demographic Characteristics Interrupted time series model for exacerbation events before and after 2015 by relevant demographic characteristics.

All age groups had an improvement over time following 2015. In the prebiologics period (2006-2015), the middle-aged group (40-64 years) had more exacerbation events than the younger and older groups, and they had greater improvements following 2015 (eTable 1 in Supplement 1 and Figure 2A). Both sexes experienced sustained reductions in annual exacerbation events, with a reversal in trend from positive preexposure slopes to negative postexposure slopes. (eTable 1 in Supplement 1 and Figure 2B).

#### By BMI

In the preexposure period, patients with a BMI of 30 or greater experienced slightly more exacerbation events compared with those with a BMI of less than 30. Both groups showed steadily decreasing trends of exacerbations after 2015 (eTable 1 in Supplement 1 and Figure 2C).

#### By Insurance Type and Geographical Residence in the Inner City

All patients, regardless of insurance, exhibited significant improvements in exacerbations. Patients living in the inner city did not demonstrate significant improvements in exacerbations immediately after the exposure period, unlike those living outside the inner city, but showed significant improvements over time (eTable 1 in Supplement 1 and Figure 2E-F).

#### By Severity

**Figure 3.**
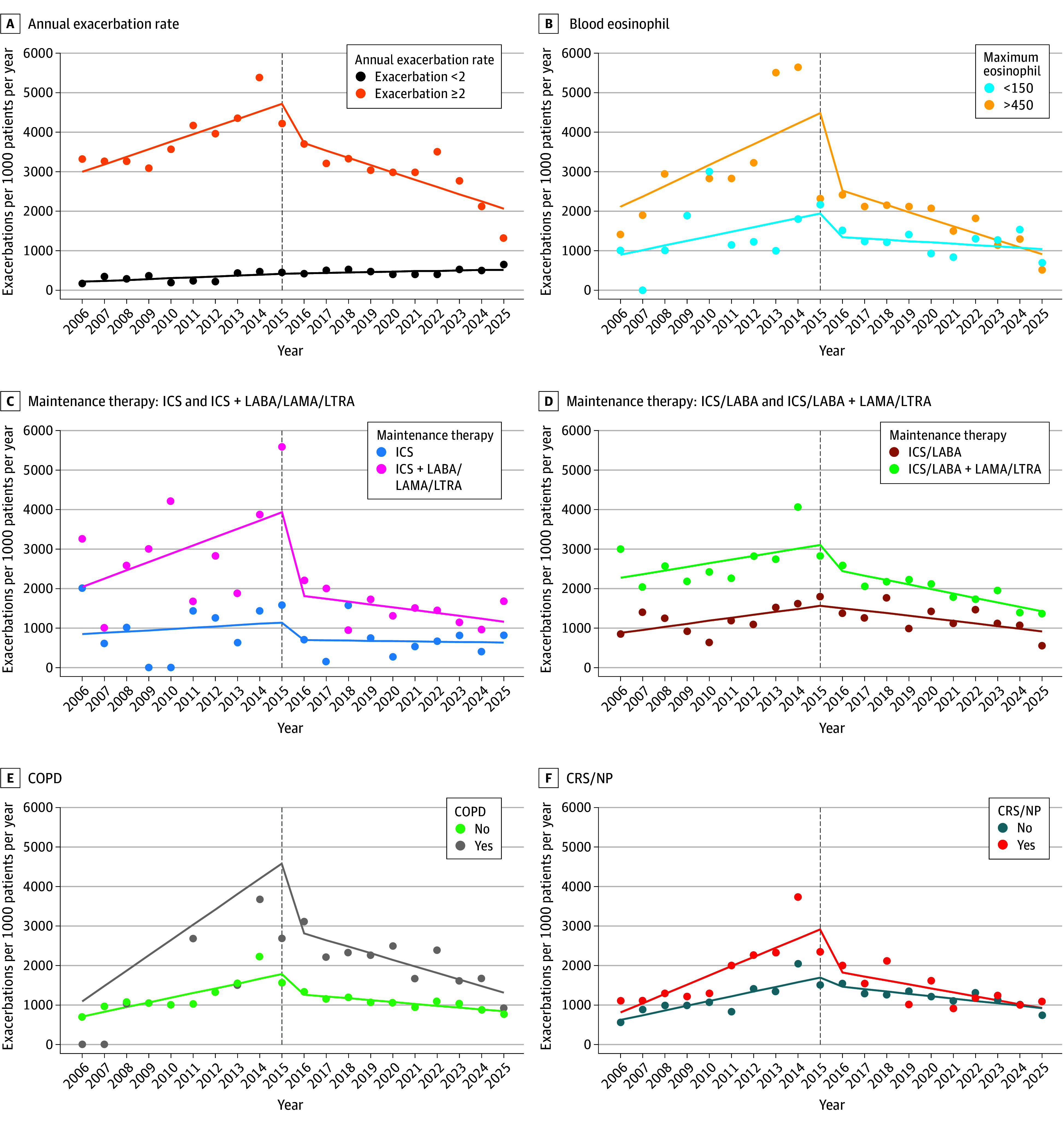
Line Graphs of the Interrupted Time Series Model for Exacerbation Events Before and After 2015 by Relevant Clinical Characteristics Interrupted time series model for exacerbation events before and after 2015 by clinical characteristics stratified by annual exacerbation rate (<2 vs ≥2 events; A), maximum baseline eosinophil count (<150 vs ≥450 cells/μL; B), maintenance therapy (inhaled corticosteroids [ICS] alone vs ICS with long-acting β-agonists [LABA], long-acting muscarinic agonists [LAMA], or leukotriene receptor agonists [LTRA]; C), maintenance therapy of ICS and LABA alone vs ICS and LABA with LAMA or LTRA; D), presence of chronic obstructive pulmonary disease, (COPD; E), and presence of chronic rhinosinusitis (CRS) with or without nasal polyps (NP; F).

Among patients with 2 or more baseline exacerbations, the preexposure trend showed a progressive increase in exacerbations (190.9 [95% CI, 88.5-293.3] exacerbation events per 1000 patients per year). There was a reduction in exacerbations immediately following 2015, although it was not statistically significant (–808.2 [95% CI, −1646.5 to 30.2] exacerbation events per 1000 patients per year) (eTable 1 in Supplement 1 and Figure 3A). There was also a sustained reduction in exacerbations in this subgroup over time (–374.5 [95% CI, −519.4 to −229.7] exacerbation events per 1000 patients per year). Among patients with milder severity, there was a slight increase over time in the preexposure period (22.4 [95% CI, 2.8 to 42.0] exacerbation events per 1000 patients per year), and neither the immediate decrease nor the sustained postbiologics trend was statistically significant (eTable 1 in Supplement 1).

#### By Eosinophil Count

Patients with BEC levels of 450 cells/μL or greater exhibited significant reduction in exacerbation events post-biologics (−1799.8 [95% CI, −3555.3 to −44.3] exacerbation events per 1000 patients per year), and a sustained downward trend in annual exacerbation events after 2015 (−443.4 [95% CI, −746.7 to −140.0] exacerbation events per 1000 patients per year). There was no significant drop in exacerbation events or sustained reductions among those with a BEC less than 150 cells/μL (eTable 1 in Supplement 1 and Figure 3B).

#### By Use of Additional Maintenance Therapies

Patients who used other maintenance therapies in addition to ICS exhibited rising but statistically nonsignificant exacerbation rates before 2015 (209.3 [95% CI, −7.1 to 425.6] exacerbation events per 1000 patients per year). There was an immediate and statistically significant decline in 2015 (–2041.2 [95% CI, −3812.1 to −270.4] exacerbation events per 1000 patients per year), followed by a continuous but statistically nonsignificant reduction (–281.5 [95% CI, −587.4 to 24.5] exacerbation events per 1000 patients per year) (eTable 1 in Supplement 1 and Figure 3C). Those who did not use additional maintenance therapy showed stable exacerbation rates over the study period (eTable 1 in Supplement 1). Patients taking ICS with long-acting β-agonists and taking other maintenance therapy in addition to ICS and long-acting β-agonists had sustained decreases in the exacerbation rates (eTable 1 in Supplement 1 and Figure 3D).

#### By Comorbid COPD or Chronic Rhinosinusitis With or Without Nasal Polyposis

Patients without concomitant COPD had significant reductions in their exacerbation events compared to those with COPD, although the magnitude of change in slope was smaller (−167.0 [95% CI, −227.8 to −106.2] exacerbation events per 1000 patients per year for patients without COPD and −553.6 [95% CI, −1170.4 to 63.1] exacerbation events per 1000 patients per year for patients with COPD) (eTable 1 in Supplement 1 and Figure 3E). Patients with concomitant chronic rhinosinusitis with or without nasal polyposis (CRS/NP) demonstrated greater improvements over time in exacerbation events compared with those without CRS/NP (−334.8 [95% CI, −467.0 to −199.8] exacerbation events per 1000 patients per year for patients with CRS/NP and −178.3 [95% CI, −240.9 to −115.7] exacerbation events per 1000 patients per year for patients without CRS/NP) (eTable 1 in Supplement 1 and Figure 3F).

### Sensitivity Analyses

#### Adjusting for Changes in Population Demographics From Preexposure to Postexposure

Compared with the pre-2015 population, the post-2015 cohort was older, more likely to reside in inner-city areas, and had a higher prevalence of obesity and COPD comorbidities. In inverse probability treatment weighting analyses to account for these differences, the preexposure upward trend and postexposure reduction remained significant compared with the unadjusted estimates (eFigure and eTable 2 in Supplement 1).

#### Adjusting for the COVID-19 Pandemic, the Uptake of SMART Therapy, and Autocorrelation

After increasing the observed post-2020 exacerbation rates by 30% and 50% to account for the impact of COVID-19 and SMART therapies, respectively, the immediate (–555.2 [95% CI, −940.9 to −169.4] exacerbation events per 1000 patients per year) and sustained (–135.1 [95% CI, −201.7 to −68.5] exacerbation events per 1000 patients per year) outcomes remained statistically significant (eFigure and eTable 2 in Supplement 1). The estimated level change and slope were largely unchanged when accounting for autocorrelation (eFigure and eTable 2 in Supplement 1).

## Discussion

In this cohort study of 5269 patients spanning nearly 2 decades, we observed a significant reduction in asthma exacerbation events following the respiratory biologic boom in 2015. Prior to this period, exacerbation events demonstrated an upward trajectory, reflecting the persistent burden of uncontrolled asthma at the population level despite the availability of standard controller therapies. Following the introduction of antieosinophilic and anti-interleukin-4 receptor α therapies, however, we identified both an immediate reduction and a sustained decline in exacerbation events, suggesting durable benefits of biologic therapies over time and supporting evidence on the estimated effectiveness of these therapies. The observed population-level reduction in exacerbation events was greatest among patients with 2 or more baseline exacerbation events per year while neither the immediate decrease nor the sustained postexposure trend was significant among patients with fewer than 2 exacerbations annually. This differential impact supported our hypothesis that the post-2015 changes in exacerbations were most likely related to the introduction of additional biologics onto the market and that their use and effectiveness are predominantly among patients with more severe disease.

This greater estimated effectiveness of biologics among patients with high baseline exacerbation rates alignd with previous studies on the effectiveness of biologics.^24,25,26,27^ Similarly, patients with BEC levels of 450 cells/μL or greater exhibited greater reductions in exacerbation rates compared with those with BEC levels of less than 150 cells/μL, which was unsurprising given that most biologics target eosinophilic inflammation. Our results are aligned with previous cohort studies indicating that biologic responders were more likely to have higher BECs.^25,28^ We also found greater reductions in exacerbation events in patients with comorbid COPD and CRS. Asthma, COPD, and CRS are interrelated through shared inflammatory pathways, and comorbid CRS and COPD could worsen asthma control.^29,30,31^ The greater reductions in exacerbation events among patients with these comorbidities suggests that biologics may offer greater benefit to patients with overlapping airway diseases who might have had poor control with conventional therapies.^32^

In sex-stratified analysis, we observed similar trends among both males and females consistent with Baptist et al,^26^ a large multicenter observational study of subspecialist-treated US adults with severe asthma. In age-stratified analyses, the middle-aged group (40-64 years) exhibited the highest exacerbation rates during the prebiologics period, consistent with prior epidemiological studies showing increased asthma morbidity in midlife, and showed greater improvements on these biologics.^33,34^ Likewise, although patients with severe asthma were predominantly with overweight or obesity,^35,36^ and individuals with a BMI of 30 or greater in this cohort exhibited slightly higher annual exacerbation events during the prebiologics period compared with those with a BMI less than 30, both groups showed reductions in exacerbation events after 2015 and a convergence in exacerbations between both groups. This postbiologic convergence or reduction of the outcome gap between low-risk and high-risk groups was also seen in various subgroup analyses, including when comparing individuals by exacerbation burden, by BEC, by the presence or absence of COPD and CRS/NP, and by smoking status, and suggests that biologics may mitigate or eliminate differences in outcomes between asthma patients of distinct disease severity.

While previous studies have reported that patients with asthma who had public insurance were less likely than those with private insurance to be prescribed biologics and less likely to initiate and continue therapy when prescribed,^37,38,39^ both privately and publicly insured patient groups exhibited marked improvements in exacerbation events after 2015. This divergence from prior reports might be explained by the nature of our study cohort, which was drawn from a single medical center serving a relatively homogeneous patient population who are mostly privately insured with access to specialty asthma care, thereby potentially masking insurance-related disparities.

Residence in inner-city environments had been consistently associated with worse asthma outcomes, driven by a combination of environmental exposures related to urbanization and other socioeconomic factors.^40^ In our study, patients residing in inner-city areas demonstrated persistently higher exacerbation rates over time compared with those living outside the inner city. Nonetheless, these patients also experienced a significant reduction in exacerbation events after 2015, suggesting that biologic therapies might confer meaningful benefit even in populations facing substantial environmental and social disadvantages.

After adjusting for potential confounding factors, the estimated reduction in exacerbations post-2015 was even larger, suggesting that the introduction of biologics was associated with greater reductions in exacerbations than indicated by the unadjusted analysis. Our results also remained consistent after adjusting for the impact of the COVID-19 pandemic and the improved uptake of ICS-formoterol as reliever and maintenance therapy.

### Strengths and Limitations

This large-scale cohort study enabled assessment of temporal trends before and after the introduction of asthma biologics. The extensive subgroup analyses provided insights into heterogeneity of treatment impact across biologically and socially relevant groups.

This study also has limitations. As an observational study, causal inference cannot be definitively established. Although the timing and magnitude of the observed reduction in exacerbations implicated the introduction of biologic therapies as the cause, other concurrent changes in asthma management or health policy may have contributed. Additionally, some portion of the observed change could reflect a natural leveling off or regression to the mean. Nevertheless, subgroup analyses stratified by disease severity, along with sensitivity analyses supported our conclusion that the post-2015 improvements were largely attributable to the biologics. Second, we used *ICD-9* and *ICD-10* codes to identify patients with moderate to severe asthma, which might be subject to misclassification. To address this, we conducted subgroup analyses based on more objective indicators of severity and our results remained consistent. Third, results were derived from a single urban center potentially limiting generalizability to other settings, including rural populations or cohorts with different demographic and insurance profiles. Fourth, the observed reduction in exacerbations should be interpreted as a temporal association rather than a direct estimate of treatment effectiveness because this was a population-level analysis and not all patients received biologic therapy. Fifth, while exacerbation events were a clinically meaningful outcome, additional metrics such as lung function, quality of life, and health care utilization were not captured.

## Conclusions

This cohort study using interrupted time series analysis demonstrated that the introduction of asthma biologic therapies in 2015 was associated with both immediate and sustained reductions in exacerbation events at the population level. The greatest benefits were observed among patients with severe disease, elevated eosinophil counts, and comorbid airway conditions, narrowing the outcome gap between patients with mild and moderate to severe asthma. These findings highlighted the effectiveness of biologics in reducing asthma morbidity and their potential to reduce disparities in asthma-related outcomes.^41^

## Notes

Supplement 1. eTable 1. Coefficients for ITS Models in Subgroup Analysis eFigure. Unadjusted and Sensitivity Analyses (Adjusting for Confounding, COVID-19/SMART Adoption, and Autocorrelation up to a 3-Year Span) eTable 2. Coefficients for ITS Models in Sensitivity Analyses (IPTW, COVID, SMART, Autocorrelation)

Supplement 2. Data Sharing Statement
