# Supplementary material for: Population-Level Trends in Asthma Exacerbations After Introduction of Respiratory Biologics
Source: JAMA Netw Open. 2026 Jun 24;9(6):e2620272. doi: 10.1001/jamanetworkopen.2026.20272 (PMC13294782; doi:10.1001/jamanetworkopen.2026.20272)
Supplement: Supplement 1. — eTable 1. Coefficients for ITS Models in Subgroup Analysis eFigure. Unadjusted and Sensitivity Analyses (Adjusting for Confounding, COVID-19/SMART Adoption, and Autocorrelation up to a 3-Year Span) eTable 2. Coefficients for ITS Models in Sensitivity Analyses (IPTW, COVID, SMART, Autocorrelation) [file jamanetwopen-e2620272-s001.pdf]

Supplemental Online Content

Tu YF, Stein DW, Akenroye A. Population-level trends in asthma exacerbations after introduction of respiratory biologics. *JAMA Netw. Open*. 2026;9(6):e2620272. doi:10.1001/jamanetworkopen.2026.20272

eTable 1. Coefficients for ITS Models in Subgroup Analysis

eFigure. Unadjusted and Sensitivity Analyses (Adjusting for Confounding, COVID-19/ SMART Adoption, and Autocorrelation up to a 3-Year Span)

eTable 2. Coefficients for ITS Models in Sensitivity Analyses (IPTW, COVID, SMART, Autocorrelation)

This supplemental material has been provided by the authors to give readers additional information about their work.

© 2026 Tu YF et al. *JAMA Network Open*.

eTable 1. Coefficients for ITS Models in Subgroup Analysis						
	Before	*P*-value	Immediate	*P*-value	After	*P*-value
Baseline exacerbation events						
≥ 2/year	190.9 (88.5 to 293.3)	.001	-808.2 (-1646.5 to 30.2)	.06	-374.5 (-519.4 to -229.7)	<.001
< 2/year	22.4 (2.8 to 42.0)	.03	1.1 (-159.3 to 161.5)	.99	-11.6 (-39.3 to 16.1)	.39
Use of other maintenance therapies in addition to ICS						
Yes	209.3 (-7.1 to 425.6)	.06	-2041.2 (-3812.1 to -270.4)	.03	-281.5 (-587.4 to 24.5)	.07
No	32.5 (-102.6 to 167.5)	.62	-438.9 (-1544.4 to 666.6)	.41	-38.8 (-229.8 to 152.2)	.67
Use of other maintenance therapies in addition to ICS/LABA						
Yes	92.4 (0 .0 to 184.9)	0.05	-549.3 (-1306.1 to 207.4)	0.14	-205.3 (-336.0 to -74.6)	0.004
No	76.2 (8.9 to 143.4)	0.03	6.2 (-544.2 to 556.6)	0.98	-141.6 (-236.7 to -46.5)	0.006
Baseline maximum eosinophil count (cells/mcl)						
<150	116.1 (-21.5 to 253.6)	.09	-569.4 (-1695.3 to 556.5)	.30	-149.2 (-343.8 to 45.3)	.12
≥ 450	264.8 (50.4 to 479.3)	.02	-1799.8 (-3555.3 to -44.3)	.05	-443.4 (-746.7 to -140.0)	.007
Age in years						
18-39	110.0 (41.9 to 178.1)	.003	175.7 (-381.7 to 733.1)	.51	-223.4 (-319.7 to -127.1)	<.001
40-64	157.7 (76.2 to 239.3)	<.001	-513.2 (-1180.9 to 154.4)	.12	-239.6 (-355.0 to -124.3)	<.001
≥65	128.6 (41.9 to 1085.0)	.006	-269.7 (-979.3 to 439.9)	.43	-178.0 (-300.5 to -55.4)	.007
Sex						
Female	129.2 (72.6 to 185.8)	<.001	-324.7 (-787.9 to 138.4)	.16	-192.9 (-272.9 to -112.9)	<.001
Male	154.6 (89.7 to 219.6)	<.001	-352.0 (-883.9 to 179.8)	.18	-228.7 (-320.5 to -136.8)	<.001
Body mass index, BMI (kg/m^2)						

© 2026 Tu YF et al. *JAMA Network Open*.

<30	132.9 (96.3 to 169.5)	<.001	-361.4 (-660.8 to -62.0)	.02	-188.0 (-239.7 to -136.3)	<.001
≥ 30	86.6 (15.9 to 157.2)	.02	-430.8 (-1008.9 to 147.3)	.13	-160.3 (-260 .2 to -60.4)	.004
Smoking						
Never	173.5 (134.6 to 212.4)	<.001	-495.5 (-813.9 to -177.0)	.005	-234.8 (-289.9 to -179.8)	<.001
Current/Former	173.9 (63.4 to 284.4)	0.004	-507.4 (-1411.7 to 397.0)	.25	-220.9 (-377.2 to -64.7)	.009
Insurance type						
Public	133.6 (71.6 to 195.5)	<.001	-216.9 (-724.3 to 290.4)	.38	-214.2 (-301.8 to -126.5)	<.001
Private	143.6 (98.4 to 188.8)	<.001	-415.5 (-785.4 to -45.6)	.03	-197.5 (-261.4 to -133.6)	<.001
Residence						
Inner City	171.5 (24.3 to 318.7)	.03	-379.9 (-1584.7 to 824.9)	.51	-276.1 (-484.3 to -68.0)	.01
Others	133.4 (92.2 to 174.7)	<.001	-340.6 (-678.2 to -3.1)	.05	-193.6 (-251.9 to -135.3)	<.001
COPD						
Yes	387.3 (-52.1 to 826.8)	.08	-1603.54 (-5146.5 to 1939.4)	.35	-553.6 (-1170.4 to 63.1)	.08
No	120.0 (77.0 to 163.0)	<.001	-474.2 (-825.9 to -122.6)	.01	-167.0 (-227.8 to -106.2)	<.001
CRS/NP						
Yes	233.9 (139.1 to 328.6)	<.001	-997.2 (-1772.9 to -221.4)	.02	-334.8 (-467.0 to -199.8)	<.001
No	118.3 (74.0 to162.6)	<.001	-170.9 (-533.4 to 191.6)	.33	-178.3 (-240.9 to -115.7)	<.001

*Abbreviations: COPD, chronic obstructive lung diseases; CRS/NP, chronic rhinosinusitis with or without nasal polyposis; ICS, inhaled corticosteroids; ITS, interrupted time series; LABA, long-acting β_2-agonists; LAMA, long-acting muscarinic antagonists; LTRA, leukotriene receptor antagonist.

© 2026 Tu YF et al. *JAMA Network Open*.

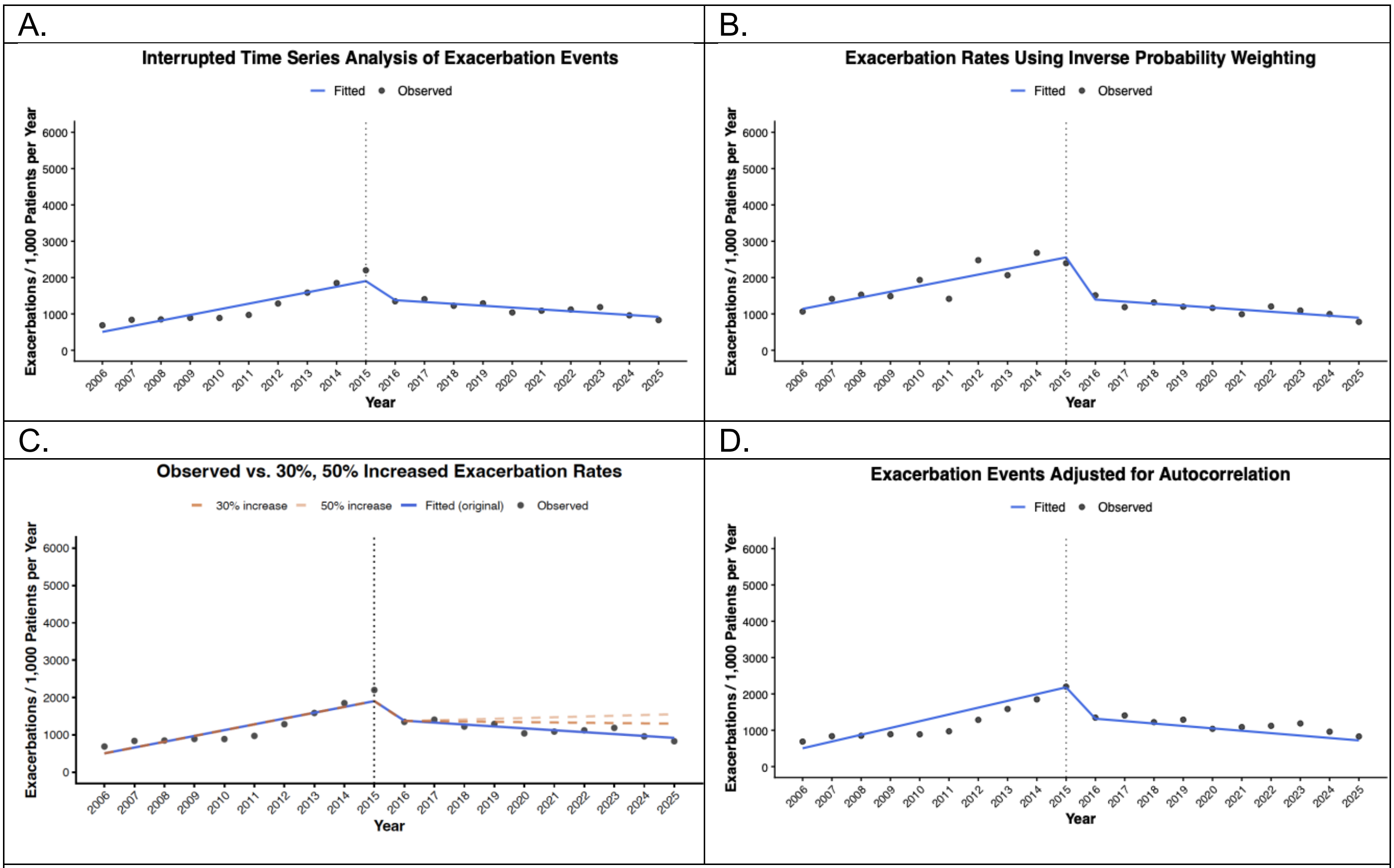

eFigure. Unadjusted and Sensitivity Analyses (adjusting for confounding, COVID-19/SMART adoption, and autocorrelation up to a 3-year span).

© 2026 Tu YF et al. *JAMA Network Open*.

eTable 2. Coefficients for **ITS Models in Sensitivity Analyses (IPTW, COVID, SMART, autocorrelation)**

**Unadjusted coefficients (Panel A)**		
Covariate	Coefficients (95% CI)	*P* value
Before	155.4 (117.7 to 193.2)	<.001
Immediate	-474.1 (-783.2 to -165.0)	.005
After	-206.5 (-259.9 to -153.0)	<.001
**Adjusting** with inverse probability weighting **(Panel B)**		
Before	157.5 (108.0 to 206.9)	<.001
Immediate	-1105.3 (-1510.1 to -700.6)	<.001
After	-213.0 (-283.0 to 143.1)	<.001
**Assuming COVID pandemic/SMART led to 30% reduction in exacerbations post-2020**		
Before	155.4 (114.2 to 196.7)	<.001
Immediate	-522.7 (-860.3 to -185.1)	0.005
After	-163.6 (-222.0 to -105.3)	<.001
**Assuming COVID pandemic/SMART led to 50% reduction in exacerbations post-2020**		
Before	155.4 (108.3 to 202.6)	<.001
Immediate	-555.2 (-940.9 to -169.4)	0.008
After	-135.1 (-201.7 to -68.5)	<.001
**Adjusting for autocorrelation up to 3-year span**		
Before	186.4 (97.6 to 275.3)	.08
Immediate	-797.6 (-1033.4 to -561.8)	<.001
After	-252.8 (-411.0 to -94.5)	.007

*Abbreviations: CI, confidence interval; ITS, interrupted time series; SMART, synchronized maintenance and reliever therapy with inhaled corticosteroids-formoterol.

© 2026 Tu YF et al. *JAMA Network Open*.
