# Supplementary material for: Population-Level Trends in Asthma Exacerbations After Introduction of Respiratory Biologics
Source: JAMA Netw Open. 2026 Jun 24;9(6):e2620272. doi: 10.1001/jamanetworkopen.2026.20272 (PMC13294782; doi:10.1001/jamanetworkopen.2026.20272)
Supplement: Supplement 2. — Data Sharing Statement [file jamanetwopen-e2620272-s002.pdf]

Data Sharing Statement

Tu. Population-Level Trends in Asthma Exacerbations After Introduction of Respiratory Biologics. *JAMA Netw Open*. Published June 24, 2026.
doi:10.1001/jamanetworkopen.2026.20272

Data
Data available: No

Additional Information
Explanation for why data not available: No new data was generated for this study. The data used can be requested through the MGB IRB after establishment of appropriate data use agreement.
